# Taxonomy and phylogeny of *Dichostereum* (Russulales), with descriptions of three new species from southern China

**DOI:** 10.3897/mycokeys.40.28700

**Published:** 2018-10-10

**Authors:** Shi-Liang Liu, Shuang-Hui He

**Affiliations:** 1 Institute of Microbiology, Beijing Forestry University, Beijing 100083, China Beijing Forestry University Beijing China

**Keywords:** Amyloid basidiospores, corticioid fungi, dichohyphae, Peniophoraceae, *
Vararia
*

## Abstract

Nine species of *Dichostereum* were subjected to phylogenetic analyses, based on a combined dataset of ITS1-5.8S-ITS2-nrLSU-*tef1* sequences. The morphology of specimens collected from China and Australia were studied. Three species, *D.austrosinense*, *D.boidinii* and *D.eburneum*, collected from southern China, are described and illustrated as new to science, based on the morphological and molecular evidence. *Dichostereumaustrosinense* is characterised by the relatively large gloeocystidia (80–130 × 8–15 µm) and basidiospores (7.3–8 µm in diam.) with large warts and crests. *Dichostereumboidinii* is distinguished by its thick basidiomata and relatively small basidiospores (5.5–6.5 µm in diam.) with large warts and crests. *Dichostereumeburneum* is unique in having pale basidiomata growing on bark of living *Castanopsis*, abundant crystals in the context and basidiospores with dense and large ornamentations. A key to the 5 species of *Dichostereum* in China is given.

## Introduction

*Dichostereum* Pilát, typified with *D.durum* (Bourdot & Galzin) Pilát, is a small and well-delimited corticioid genus in Russulales. It is characterised by resupinate basidiomata with smooth or grandinioid hymenophore, dimitic hyphal system with dextrinoid dichohyphae and clamped generative hyphae, gloeocystidia and ellipsoid or subglobose, ornamented basidiospores with a strong amyloid reaction in Melzer’s reagent (Hallenberg 1985; Bernicchia and Gorjón 2010). Previously, *Dichostereum* was placed in Lachnocladiaceae, which includes genera with dextrinoid skeletal hyphae (Reid 1965; Parmasto 1968, 1970; Hallenberg 1985). However, recent phylogenetic analyses, based on DNA sequences, showed that *Dichostereum* formed a monophyletic lineage in the Peniophoraceae clade, which includes genera with or without dextrinoid hyphae (Larsson and Larsson 2003; Miller et al. 2006; Larsson 2007).

*Dichostereum* was once treated as a subgenus of *Vararia* P. Karst. (Peniophoraceae, Russulales) by some mycologists since the two genera are very similar in morphology except that the latter has smooth basidiospores (Boidin 1967; Parmasto 1970; Lanquetin 1973). Boidin and Lanquetin (1977) emended the description of *Dichostereum* and retained it as a separate genus. Later, Boidin and Lanquetin (1980) monographed the genus and provided a key to its 11 species based on evidence of morphology, distribution and intercompatibility tests of cultures. Based on limited sampling, their results showed that *D.effuscatum* (Cooke & Ellis) Boidin & Lanq. and *D.granulosum* (Pers.) Boidin & Lanq. were widely distributed, while the other species seemed to be rather endemic (Boidin and Lanquetin 1980). Few studies on the genus have been carried out since then and many regions including East Asia need further collecting and study (Boidin et al. 1987, Boidin and Michel 1998).

Previously, two species, *Dichostereumboreale* (Pouzar) Ginns & M.N.L. Lefebvre (= *D.granulosum*) and *D.pallescens* (Schwein.) Boidin & Lanq., were reported in temperate China (Dai 2011). The species diversity of the genus in China is still unclear. In the present paper, we provide a morphological and phylogenetic study of the genus based on specimens mostly collected from southern China. This is part of an ongoing study of the corticioid fungi of the Russulales in China.

## Materials and methods

### Morphological studies

Voucher specimens were deposited in the herbaria of Beijing Forestry University, Beijing, China (**BJFC**) and in the Centre for Forest Mycology Research, U.S. Forest Service, Madison, USA (**CFMR**). Freehand sections were made from dried basidiomata and mounted in 2% (w/v) potassium hydroxide (KOH), 1% phloxine (w/v) or Melzer’s reagent. Microscopic examinations were carried out with a Nikon Eclipse 80i microscope (Nikon Corporation, Japan) at magnifications up to 1000×. Drawings were made with the aid of a drawing tube. All measurements were carried out with sections mounted in Melzer’s reagent. Ornamentations were excluded from the measurements of basidiospores. Scanning electron micrographs (SEM) were taken with a JEOL JSM-6700F microscope (JEOL, Japan). Dried specimens were mounted directly in gold and platinum and examined and photographed at 10.0 kV. Colour names and codes follow Kornerup and Wanscher (1978). Herbarium code designations are from Index Herbariorum (Thiers, continuously updated).

### DNA extraction and sequencing

The CTAB plant genome rapid extraction kit DN14 (Aidlab Biotechnologies Co. Ltd, Beijing) was used for DNA extraction and PCR amplification from dried specimens or cultures. The ITS, partial nrLSU and *tef1* markers were amplified with the primer pairs ITS5/ITS4 (White et al. 1990), LR0R/LR7 (Vilgalys and Hester 1990) and 983F/1567R (Rehner and Buckley 2005), respectively. The PCR procedures followed Dai and He (2016). DNA sequencing was performed at Beijing Genomics Institute and the sequences were deposited in GenBank (Benson et al. 2018). The sequence quality control followed Nilsson et al. (2012). BioEdit v.7.0.5.3 (Hall 1999) and Geneious v.11.1.15 (Kearse et al. 2012) were used for chromatogram check and contig assembly.

### Phylogenetic analyses

The molecular phylogeny was inferred from a combined dataset of ITS1-5.8S-ITS2-nrLSU-*tef1* sequences of representative members of Peniophoraceae*sensu*Larsson (2007) (Table 1). *Echinodontiumtinctorium* (Ellis & Everh.) Ellis & Everh. was selected as the outgroup (Liu et al. 2017a). The sequences of the three markers (ITS, nrLSU and *tef1*) were aligned separately using MAFFT v.7 (Katoh et al. 2017, http://mafft.cbrc.jp/alignment/server/) with the G-INS-i iterative refinement algorithm and optimised manually in BioEdit v.7.0.5.3. The programme Gblocks v.0.91b (Castresana 2000) was then used to exclude poorly aligned positions of the ITS alignment. The separate alignments were concatenated using Mesquite v.3.5.1 (Maddison and Maddison 2018). The combined alignments were deposited in TreeBase (http://treebase.org/treebase-web/home.html, submission ID: 23332).

**Table 1.** Species and sequences used in the phylogenetic analyses. Newly generated sequences are set in bold. Holotypes are marked with *.

Taxa	Voucher	Locality	ITS	nrLSU	*tef1*
* Asterostroma bambusicola *	He 4132	Thailand	KY263865	KY263871	**MH669240**
* A. cervicolor *	He 4020	China	KY263859	KY263869	–
* Baltazaria eurasiaticogalactina *	CBS 666.84	France	–	AY293211	–
* B. octopodites *	FLOR 56449	Brazil	MH260025	MH260047	–
* Dichostereum austrosinense *	He 4871*	China	**MH538317**	**MH538334**	–
	He 4316	China	**MH538316**	**MH538335**	–
	He 3551	China	**MH538314**	–	**MH550363**
* D. boidinii *	He 5026*	China	**MH538324**	**MH538330**	–
	He 1662	China	**MH538309**	–	**MH550360**
	He 4410	China	**MH538315**	**MH538331**	**MH550361**
	He 462	China	**MH538311**	–	–
	Dai 16117	China	**MH538312**	**MH538327**	**MH550362**
* D. durum *	Fungi Gallici 1985	France	AF506429	AF506429	–
* D. eburneum *	He 5374*	China	**MH538318**	**MH538337**	**MH550366**
* D. effuscatum *	GG 930915	France	AF506390	AF506390	–
	FP 101758 Sp	USA	**MH538323**	**MH538336**	**MH550367**
	CBS 516.80	USA	–	AF323739	–
* D. granulosum *	NH 7137	Canada	AF506391	AF506391	–
	FP 133479 Sp	USA	**MH538321**	**MH538333**	**MH550368**
	He 1887	China	**MH538313**	**MH538332**	–
* D. pallescens *	Kropp 2	USA	**MH538320**	**MH538326**	**MH550365**
	CBS 717.81	USA	–	AF518614	–
	He 3266	China	**MH538310**	**MH538325**	**MH550364**
D. aff. pallescens	KHL 10258	Puerto Rico	AF506428	AF506428	–
* D. rhodosporum *	Dai 18625A	Australia	**MH538319**	**MH538329**	**MH550370**
* D. sordulentum *	FP 11735 Sp	USA	**MH538322**	**MH538328**	**MH550369**
* Duportella tristicula *	He 4775	China	**MH669235**	**MH669239**	**MH669245**
* Echinodontium tinctorium *	HHB 12866 Sp	USA	KY172888	KY172903	**MH550371**
* Gloiothele lactescens *	EL 8-98	Sweden	AF506453	AF506453	–
* G. lamellosa *	KHL11031	Venezuela	AF506454	AF506454	–
Lachnocladium cf. brasiliense	CALD 161213-1	Brazil	MH260037	MH260055	–
* L. schweinfurthianum *	KM 49740	Cameroon	MH260033	MH260051	–
*L.* sp.	KHL10556	Jamaica	AF506461	AF506461	–
* Parapterulicium subarbusculum *	FLOR 56456	Brazil	MH260026	MH260026	–
	FLOR 56459	Brazil	MH260027	MH260049	–
* Peniophora polygonia *	He 3668	China	**MH669233**	**MH669237**	**MH669243**
* P. rufa *	He 2788	China	**MH669234**	**MH669238**	**MH669244**
* Scytinostroma portentosum *	EL11-99	Sweden	AF506470	AF506470	–
* Vararia investiens *	He 2104	USA	–	**MH669236**	**MH669242**
	FP 151122	USA	**MH971976**	**MH971977**	–
* Vesiculomyces citrinus *	He 3716	China	KY860369	KY860429	**MH669241**

For both Maximum Likelihood (ML) and Bayesian Inference (BI), a partitioned analysis was performed with the following five partitions: ITS1, 5.8S, ITS2, nrLSU and *tef1*. The ML analysis was performed using RAxML v.8.2.10 (Stamatakis 2014) with the bootstrap values (ML-BS) obtained from 1,000 replicates and the GTRGAMMA model of nucleotide evolution. The maximum parsimony (MP) analysis was performed using PAUP* 4.0a162 (Swofford 2003). Trees were generated using 100 replicates of random stepwise addition of sequence and tree-bisection reconnection (TBR) branch-swapping algorithm with all characters given equal weight. Branch supports (MP-BS) for all parsimony analyses were estimated by performing 1,000 bootstrap replicates with a heuristic search of 10 random-addition replicates for each bootstrap replicate. The BI was performed using MrBayes 3.2.6 (Ronquist et al. 2012). The best-fit substitution model for each partitioned locus was estimated separately with jModeltest v.2.17 (Darriba et al. 2012). Four Markov chains were run for 6,000,000 generations until the split deviation frequency value was lower than 0.01. The convergence of the runs was checked using Tracer v.1.7 (Rambaut et al. 2018). Trees were sampled every 100^th^ generation. The first quarter of the trees, which represented the burn-in phase of the analyses, was discarded and the remaining trees were used to calculate Bayesian posterior probabilities (BPP) in the majority rule consensus tree. All trees were visualised in FigTree 1.4.2 (Rambaut 2014).

## Results

### Phylogenetic inference

**Figure 1.**
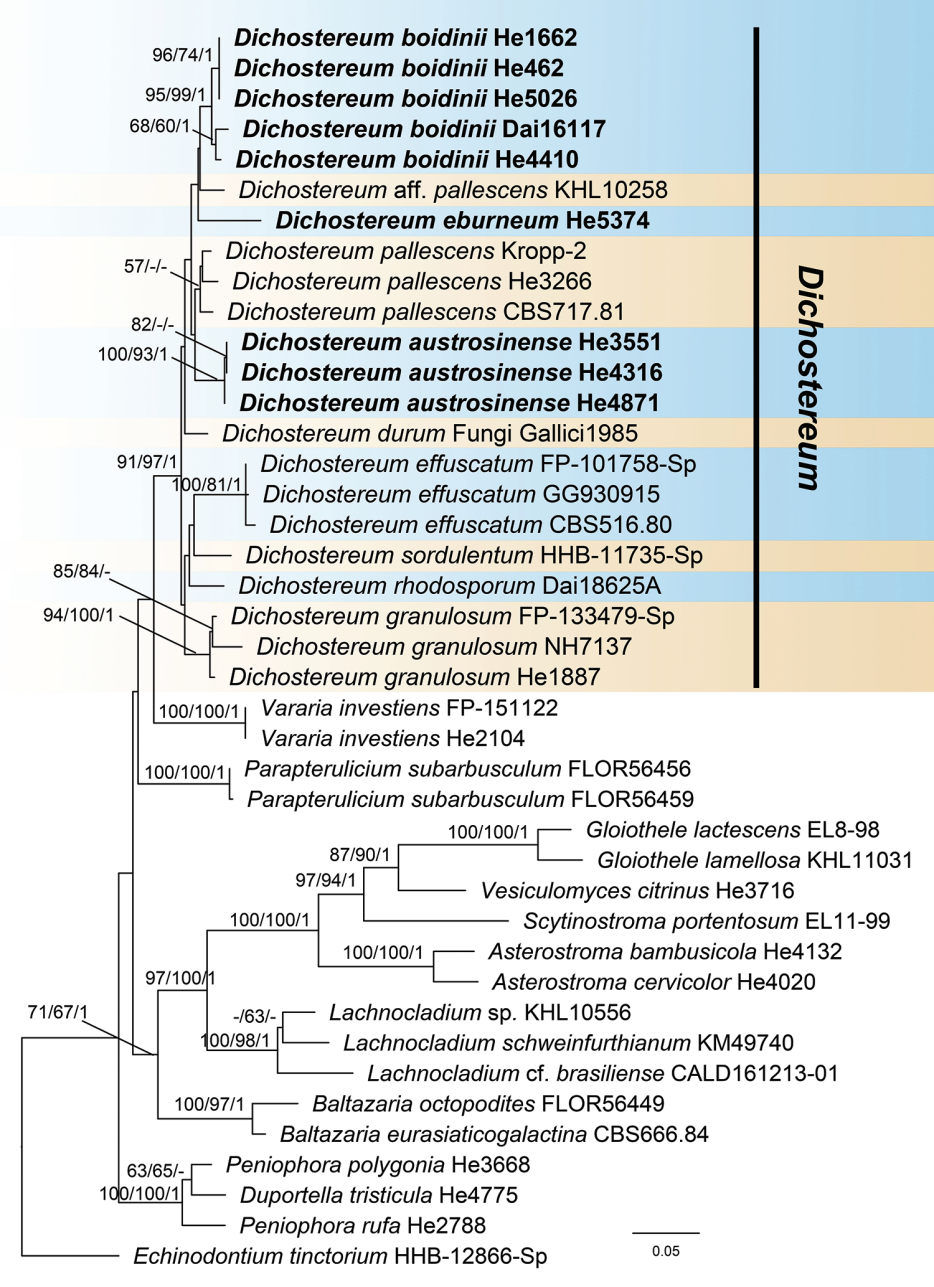
Phylogeny of *Dichostereum* and representatives of Peniophoraceae inferred from ITS-nrLSU-*tef1* sequences. Topology is from ML analysis with maximum likelihood bootstrap support values (≥50, former), parsimony bootstrap support values (≥50, middle) and Bayesian posterior probability values (≥0.95, latter) shown along the branches. Different species of *Dichostereum* are indicated as coloured blocks. The new species are set in bold. Scale bar: 0.05 nucleotide substitutions per site.

The ITS-nrLSU-*tef1* sequence dataset contained 37 ITS, 38 nrLSU and 18 *tef1* sequences from 40 samples representing 26 ingroup taxa and the outgroup (Table 1). Twenty ITS, 18 nrLSU and 18 *tef1* sequences were generated for this study. The dataset had an aligned length of 2239 characters, of which 1596 were constant, 163 variable characters were parsimony-uninformative and 480 were parsimony-informative. MP analysis yielded six most parsimonious trees. jModelTest suggested TIM2ef+G, K80+G, TPM1uf+G, TIM2+I+G and TrN+I+G to be the best-fit models of nucleotide evolution for ITS1, 5.8S, ITS2, nrLSU and *tef1* markers, respectively, for the Bayesian analysis. The average standard deviation of split frequencies of BI was 0.004704 at the end of the run. MP and BI analyses resulted in an almost identical tree topology compared to the ML analysis. Only the ML tree is shown in Fig. 1 with ML and MP bootstrap values ≥50% and Bayesian posterior probabilities ≥0.95 labelled along the branches.

In the tree (Fig. 1), the nine sampled species of *Dichostereum* formed a strongly supported clade in Peniophoraceae (ML-BS = 91, MP-BS = 97, BPP = 1.00). *Varariainvestiens* (Schwein.) P. Karst., the generic type of *Vararia*, formed a sister lineage to *Dichostereum*, but this close relationship did not receive significant support. Of the three new species, samples of *D.austrosinense* and *D.boidinii* formed two strongly supported lineages, whilst the single specimen of *D.eburneum* formed the sister taxon to *D.boidinii* and D.aff.pallescens. *Dichostereumeffuscatum* from France and USA and both *D.granulosum* and *D.pallescens* from north America and China, formed three strongly supported lineages. Single samples of *D.durum* from France, *D.rhodosporum* from Australia and *D.sordulentum* from USA formed their own distinct lineages.

### Taxonomy

#### Dichostereum austrosinense

Taxon classificationFungiRussulalesLachnocladiaceae

S.H. He & S.L. Liu sp. nov.

MB826931

Figs 2a , 3 , 6a

##### Typification.

CHINA. Guangxi Autonomous Region, Jinxiu County, Dayaoshan Nature Reserve, Shengtangshan, on fallen angiosperm trunk, 15 Jul 2017, He 4871 (holotype, BJFC 024390, ITS GenBank accession number: MH538317; isotype in CFMR).

##### Etymology.

“*austrosinense*” referring to the distribution in southern China.

##### Basidiomata.

Perennial, resupinate, effused, closely adnate, inseparable from substrates, coriaceous to soft corky, at first as irregular small patches, later confluent up to 15 cm long, 4.5 cm wide, up to 1 mm thick. Hymenophore surface smooth, greyish-orange [5B(4–5)], brownish-yellow [5C(7–8)] to light brown [6D(4–8)], not cracking; margin abrupt, concolorous or darker than hymenophore surface.

##### Microscopic structures.

Hyphal system dimitic. Context thickening, compact, composed of generative hyphae, dichohyphae, embedded basidiospores and scattered crystals. Generative hyphae rare, with clamp connections, hyaline, thin- to slightly thick-walled, 2–3 µm in diam. Dichohyphae dominant, hyaline to yellow, distinctly thick-walled, dichotomously branched with acute tips, weakly dextriniod. Catahymenium composed of dichohyphae, gloeocystidia, basidia and basidioles. Dichohyphae in this layer abundant, similar to those in the context, but strongly dextrinoid, more slender and more frequently branched, 20–50 μm across, 2–4 µm wide at lowest part. Gloeocystidia abundant, subcylindrical to subfusiform, hyaline, slightly thick-walled, with or without solidified contents, 80–130 × 8–15 µm. Basidia narrowly cylindrical, usually slightly sinuous, hyaline, thin-walled, with 4 sterigmata and a basal clamp connection, 50–80 × 5–8 µm; basidioles in shape similar to basidia, but slightly smaller. Basidiospores abundant, subglobose with a distinct apiculus, hyaline to pale yellowish-brown in KOH, thick-walled, strongly amyloid, (7–) 7.3–8 (–9) µm in diam.; walls ornamented with large warts and crests.

##### Additional specimens examined.

CHINA. Hainan Province, Lingshui County, Diaoluoshan Nature Reserve, on fallen angiosperm trunk, 17 Mar 2016, He 3551 (BJFC 022052); Jiangxi Province, Lianping County, Jiulianshan Nature Reserve, on fallen angiosperm branch, 13 Aug 2016, He 4316 (BJFC 023758).

##### Remarks.

*Dichostereumaustrosinense* is overall characterised by the relatively large gloeocystidia and basidiospores with large warts and crests. *Dichostereumpeniophoroides* (Burt) Boidin & Lanq. is similar to *D.austrosinense* but differs in having wider gloeocystidia (7–22 µm), slightly larger basidiospores (7–9 µm) with larger ornamentations and a distribution in Caribbean regions (Lanquetin 1973; Boidin and Lanquetin 1980). *Dichostereumaustrosinense* is also similar to *D.rhodosporum* (Wakef.) Boidin & Lanq. which differs in having paler basidiomata, smaller ornamentations of basidiospores and a distribution in Australia and New Zealand (Boidin and Lanquetin 1980, Figs 2 and 6).

**Figure 2.**
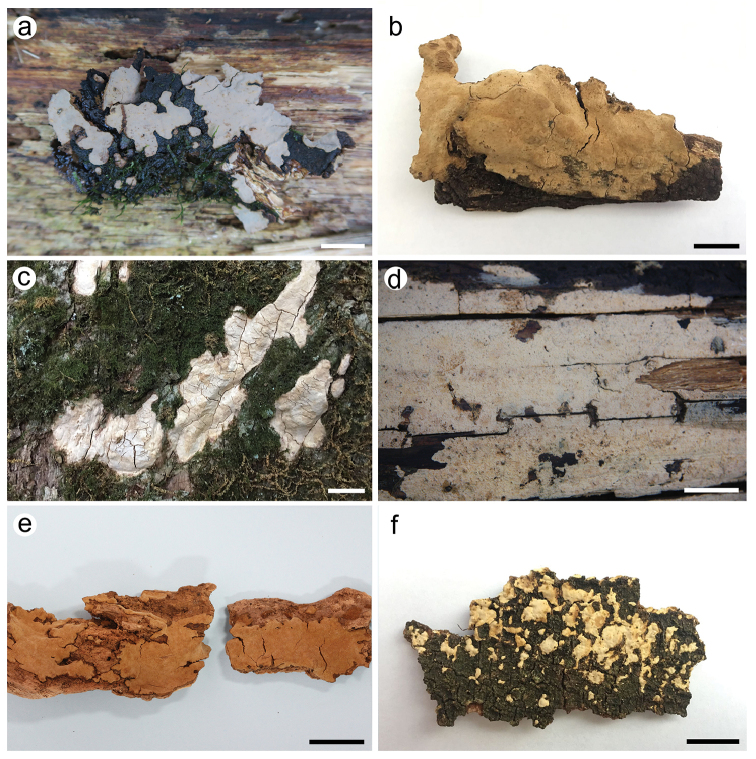
Basidiomata of *Dichostereum* species. **a***D.austrosinense* (holotype, He 4871) **b***D.boidinii* (holotype, He 5026) **c***D.eburneum* (holotype, He 5374) **d***D.granulosum* (He 1887) **e***D.pallescens* (He 3266) **f***D.rhodosporum* (Dai 18625A). Scale bar: 1 cm.

**Figure 3.**
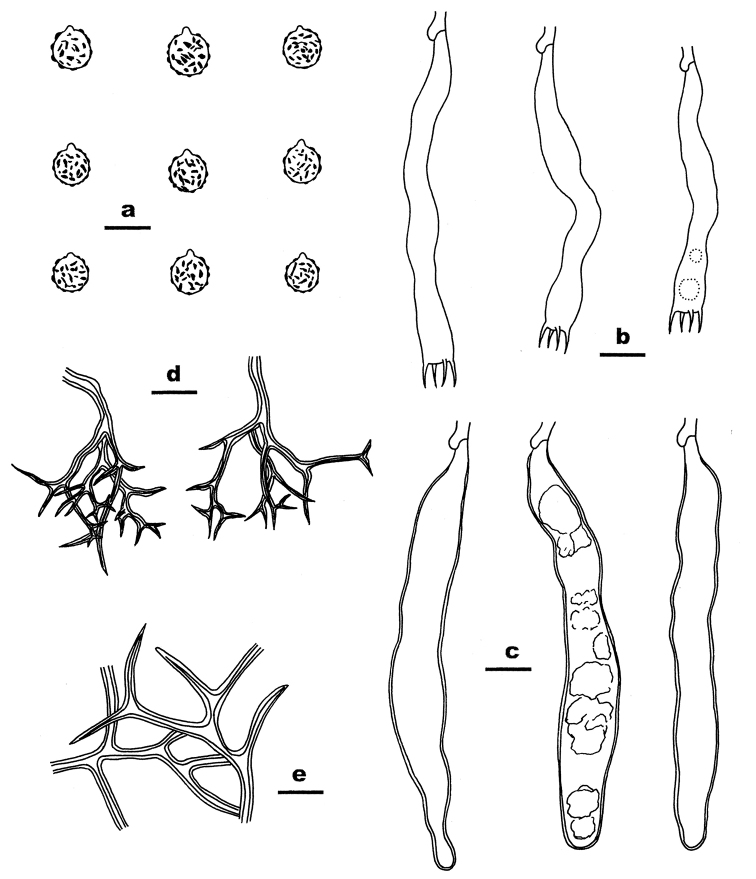
Microscopic structures of *Dichostereumaustrosinense* (drawn from the holotype). **a** Basidiospores **b** Basidia **c** Gloeocystidia **d** Dichohyphae from hymenium **e** Dichohyphae from subiculum. Scale bar: 10 µm.

#### Dichostereum boidinii

Taxon classificationFungiRussulalesLachnocladiaceae

S.H. He & S.L. Liu sp. nov.

MB826932

Figs 2b , 4 , 6b

##### Typification.

CHINA. Hubei Province, Wufeng County, Breeding base of *Magnolia*, on angiosperm stump, 14 Aug 2017, He 5026 (holotype, BJFC 024544, ITS GenBank accession number: MH538324; isotype in CFMR).

##### Etymology.

“*boidinii*” (Lat.), named to honour Dr. Jacques Boidin (Lyon, France) for his contribution to the taxonomy of *Dichostereum*.

##### Basidiomata.

Perennial, resupinate to effused-reflexed with slightly elevated margin, closely adnate, inseparable from substrates, coriaceous to soft corky, up to 8 cm long, 4 cm wide, 1.5 mm thick. Hymenophore surface smooth, greyish-orange [6B(3–4)], brownish-orange [6C(4–6)] to light brown [6D(4–6)], not cracking; margin abrupt, concolorous or darker than hymenophore surface.

##### Microscopic structures.

Hyphal system dimitic. Context thickening, compact, composed of generative hyphae, dichohyphae, embedded basidiospores and scattered crystals. Generative hyphae rare, with clamp connections, hyaline, thin-walled, 2–3 µm in diam. Dichohyphae dominant, hyaline to yellow, distinctly thick-walled, dextriniod. Catahymenium composed of dichohyphae, gloeocystidia, basidia and basidioles. Dichohyphae in this layer abundant, similar to those in the context, but strongly dextrinoid, more frequently branched with short terminal branches, 20–40 μm across, 2–4 µm wide at lowest part. Gloeocystidia abundant, fusiform to subulate, hyaline, slightly thick-walled, with solidified contents, 20–60 × 7–12 µm. Basidia subclavate to subcylindrical, hyaline, thin-walled, with 4 sterigmata and a basal clamp connection, 25–40 × 5–7 µm; basidioles in shape similar to basidia, but slightly smaller. Basidiospores subglobose with a distinct apiculus, hyaline to pale yellowish-brown in KOH, thick-walled, strongly amyloid, (5–) 5.5–6.5 (–7) µm in diam.; walls ornamented with warts and crests.

##### Additional specimens examined.

CHINA. Anhui Province, Huangshan County, Huangshan Nature Reserve, on fallen angiosperm trunk, 21 Oct 2011, He 462 (BJFC 012101); Hainan Province, Lingshui County, Diaoluoshan Nature Reserve, on rotten wood of *Dacrydium*, 13 Nov 2015, Dai 16117 (BJFC 020210); Jiangxi Province, Anyuan County, Sanbaishan Forest Park, on fallen angiosperm trunk, 15 Aug 2016, He 4410 (BJFC 023851); Yunnan Province, Kunming, Xishan Park, on angiosperm stump, 17 Jul 2013, He 1662 (BJFC 016129).

##### Remarks.

*Dichostereumboidinii* is widely distributed in southern China and mainly characterised by the thick, brownish basidiomata and relatively small basidiospores with large warts and crests. *Dichostereumpallescens* is similar to *D.boidinii* but differs in having slender dichohyphae and smaller and sparser ornamentations of basidiospores (Boidin and Lanquetin 1980, Fig. 6). *Dichostereumorientale* Boidin & Lanq. resembles *D.boidinii* by sharing short terminal branches of dichohyphae, but differs in having smaller basidiospores (5–5.5 µm in diam.) and a distribution in Africa (Boidin and Lanquetin 1980). The ornamentation of basidiospores of *D.boidinii* is similar to *D.austrosinense*, but the latter species has larger gloeocystidia, basidia and basidiospores.

**Figure 4.**
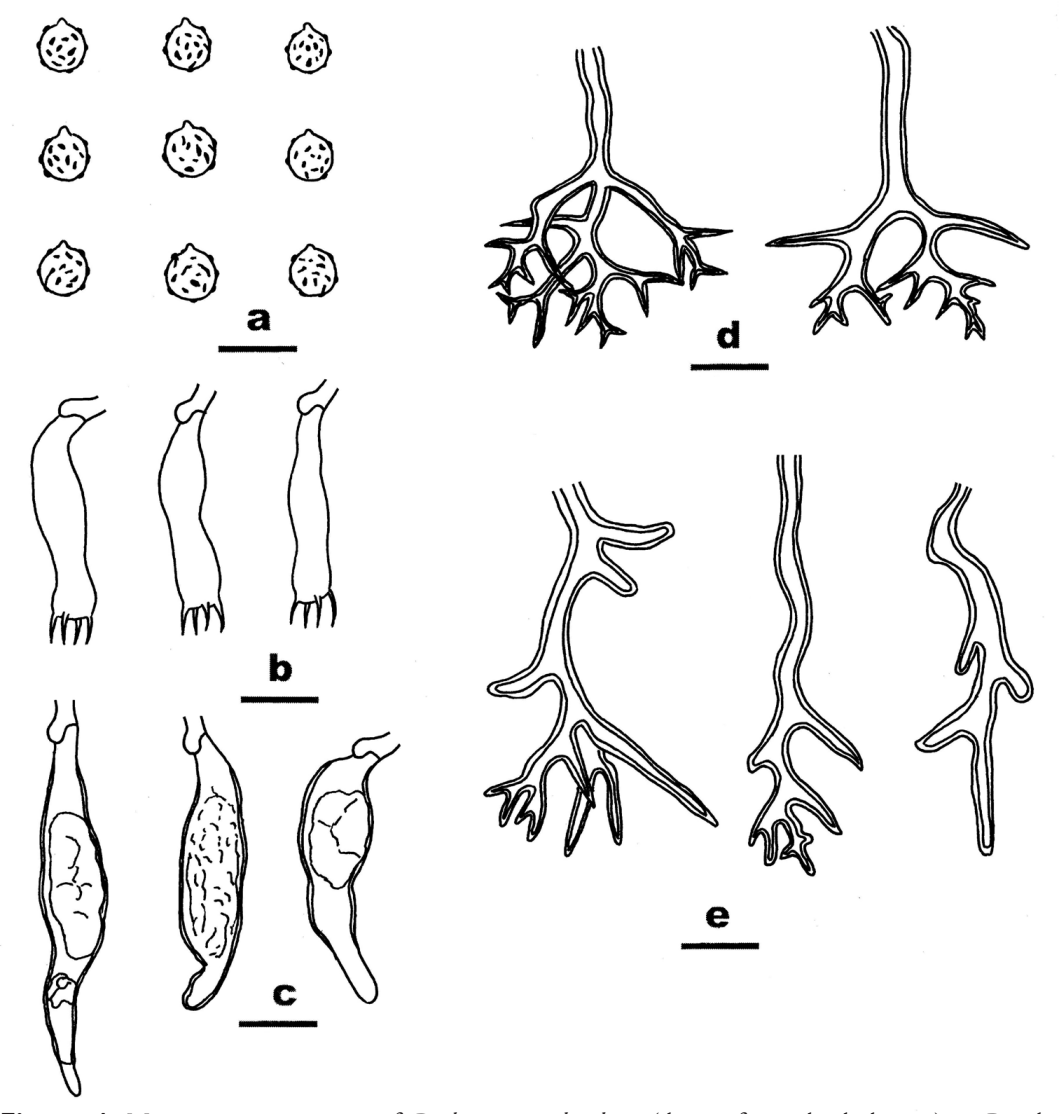
Microscopic structures of *Dichostereumboidinii* (drawn from the holotype). **a** Basidiospores **b** Basidia **c** Gloeocystidia **d** Dichohyphae from hymenium **e** Dichohyphae from subiculum. Scale bar: 10 µm.

#### Dichostereum eburneum

Taxon classificationFungiRussulalesLachnocladiaceae

S.H. He & S.L. Liu sp. nov.

MB826933

Figs 2c , 5 , 6c

##### Typification.

CHINA. Fujian Province, Wuyishan County, Wuyishan Nature Reserve, on bark of living *Castanopsis*, 6 Apr 2018, He 5374 (holotype, BJFC, ITS GenBank accession number: MH538318; isotype in CFMR).

##### Etymology.

“*eburneum*” referring to the white colour of hymenophore.

##### Basidiomata.

Perennial, resupinate, effused, closely adnate, inseparable from substrate, coriaceous, at first as irregular small patches, later confluent up to 7 cm long, 2 cm wide, 200–500 µm thick. Hymenophore surface smooth, white (5A1), orange white (5A2) to greyish-orange [5B(3–4)], cracking with age; margin thinning out, concolorous with hymenophore.

##### Microscopic structures.

Hyphal system dimitic. Context thickening, compact, composed of generative hyphae, dichohyphae, embedded basidiospores and abundant crystals. Generative hyphae rare, with clamp connections, hyaline, thin- to slightly thick-walled, 2–3 µm in diam. Dichohyphae dominant, hyaline to yellow, distinctly thick-walled, dextriniod, frequently branched, aseptate, 1–2 µm in diam. Catahymenium composed of dichohyphae, gloeocystidia, basidia and basidioles. Dichohyphae in this layer abundant, hyaline to pale yellow, distinctly thick-walled, strongly dextriniod, dichotomously branched with acute terminal tips, 15–30 μm across, 2–4 µm wide at lowest part. Gloeocystidia abundant, fusiform to subclavate, hyaline, thin-walled, with solidified contents, 20–50 × 5–10 µm. Basidia subcylindrical with basal part slightly swollen, hyaline, thin-walled, with 4 sterigmata and a basal clamp connection, 30–45 × 6–9 µm; basidioles in shape similar to basidia, but slightly smaller. Basidiospores subglobose with a distinct apiculus, hyaline to pale yellowish-brown in KOH, thick-walled, strongly amyloid, 6–7 (–8) µm in diam.; walls ornamented with dense, large warts and crests.

##### Remarks.

*Dichostereumeburneum* is characterised by the pale basidiomata on bark of living tree, the presence of abundant crystals in context and basidiospores with dense and large ornamentations. Ecologically and macroscopically, *D.eburneum* resembles *Dendrothele* Höhn. & Litsch., but the microscopic features are largely different (Nakasone and Burdsall 2011). *Dichostereumkenyense* Boidin & Lanq. is similar to *D.eburneum* by sharing the large ornamentations of basidiospores, but differs in having wider span of dichohyphae, slightly larger basidiospores (7–8 µm) and a distribution in Africa (Boidin and Lanquetin 1980).

**Figure 5.**
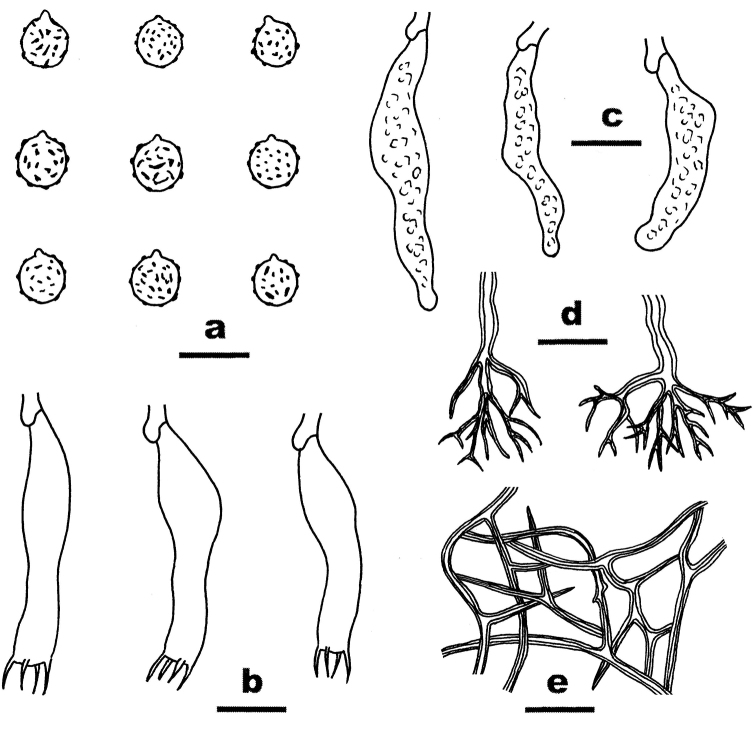
Microscopic structures of *Dichostereumeburneum* (drawn from the holotype). **a** Basidiospores; **b** Basidia **c** Gloeocystidia **d** Dichohyphae from hymenium **e** Dichohyphae from subiculum. Scale bar: 10 µm.

**Figure 6.**
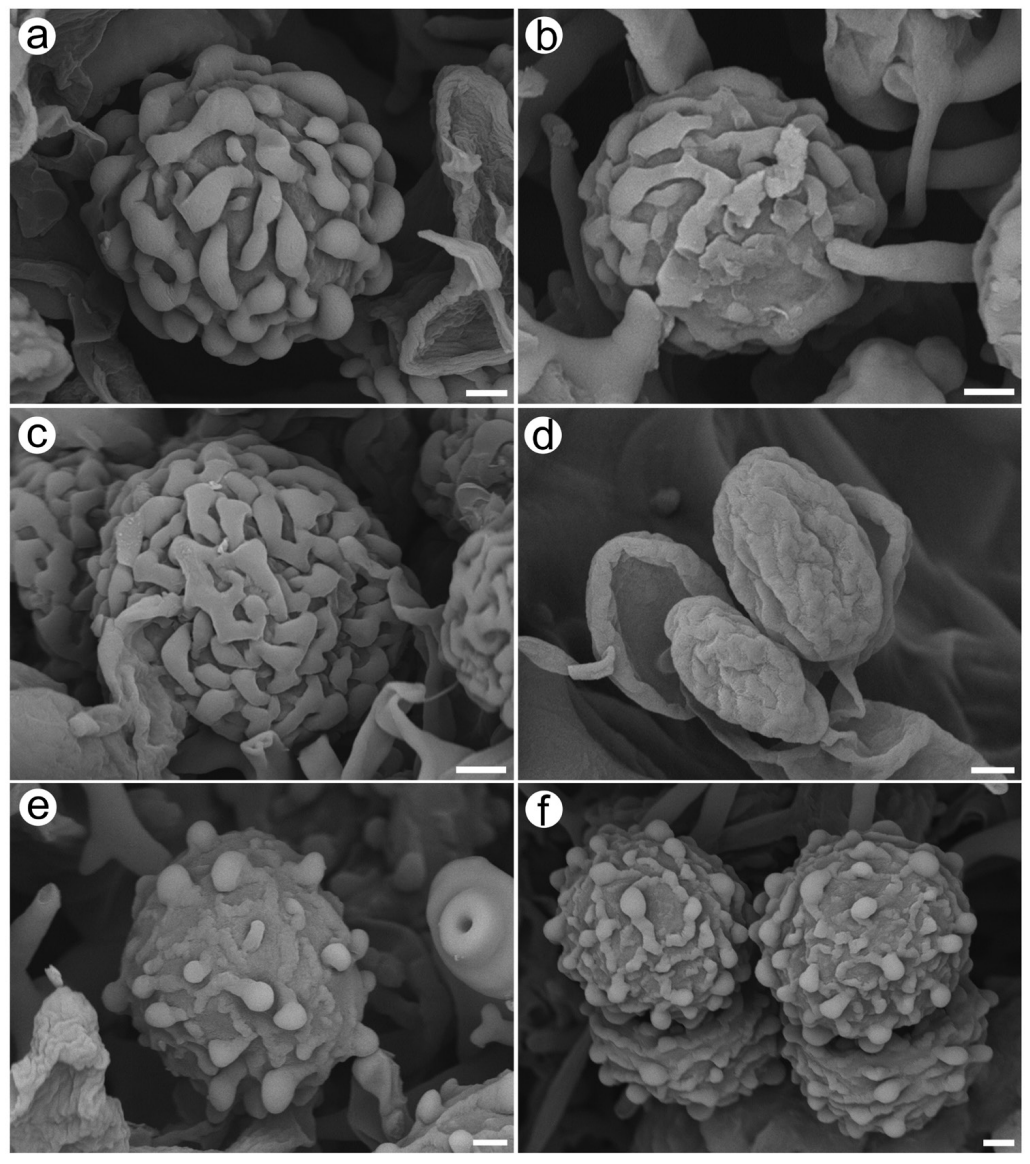
Scanning electron micrographs (SEM) of basidiospores of *Dichostereum*. **a***D.austrosinense* (holotype, He 4871) **b***D.boidinii* (holotype, He 5026) **c***D.eburneum* (holotype, He 5374) **d***D.granulosum* (He 1887) **e***D.pallescens* (He 3266) **f***D.rhodosporum* (Dai 18625A). Scale bar: 1 µm.

###### Key to 5 species of Dichostereum in China

**Table:** 

1	Hymenophore grandinioid; basidiospores ellipsoid	*** D. granulosum ***
–	Hymenophore smooth; basidiospores subglobose	**2**
2	Basidiomata white; on bark of living *Castanopsis*	*** D. eburneum ***
–	Basidiomata brownish; on dead wood	**3**
3	Gloeocystidia ≥80 μm long	*** D. austrosinense ***
–	Gloeocystidia <80 μm long	**4**
4	Basidiospores 6.5–7.5 µm in diam, ornamentation sparse	*** D. pallescens ***
–	Basidiospores 5.5–6.5 µm in diam, ornamentation dense	*** D. boidinii ***

## Discussion

To date, 14 species of *Dichostereum* have been described worldwide including the three new species in the present paper (Boidin and Lanquetin 1980). Amongst them, 5 species, *D.brevisporum* (S.S. Rattan) Boidin & Lanq. from India, *D.kenyense*, *D.orientale* and *D.ramulosum* (Boidin & Lanq.) Boidin & Lanq. from Africa and *D.peniophoroides* from Caribbean regions, were not included in the present analyses. In order to resolve the infra-generic phylogenetic relationships of *Dichostereum*, samples of these species and any additional undescribed taxa should be included.

The family Peniophoraceae*sensu*Larsson (2007) formed a strongly supported clade in Russulales and included about 15 genera (Larsson and Larsson 2003; Miller et al. 2006; Larsson 2007; Leal-Dutra et al. 2018). Except for the coralloid *Lachnocladium* Lév. and the insect symbiont *Entomocorticium* H.S. Whitney, Bandoni & Oberw., all the other genera in the family are corticioid fungi, such as *Asterostroma* Massee, *Peniophora* Cooke, *Scytinostroma* Donk and *Vararia*. However, recent molecular and morphological studies showed that two species of *Parapterulicium* Corner with coralloid basidiomata belong to Peniophoraceae in the Russulales rather than Pterulaceae of the Agaricales (Leal-Dutra et al. 2018). In the phylogenetic tree, the type species, *Parapteruliciumsubarbusculum* Corner formed a distinct lineage, while *P.octopodites* Corner is closely related to *Scytinostromagalactinum* (Fr.) Donk and its relatives. More studies on the taxonomy and phylogeny of Peniophoraceae are needed, since some large genera such as *Scytinostroma* and *Vararia* are still polyphyletic and many species are undescribed.

## Supplementary Material

XML Treatment for Dichostereum austrosinense

XML Treatment for Dichostereum boidinii

XML Treatment for Dichostereum eburneum
